# Polyphenols Regulate the Activity of Endocrine-Disrupting Chemicals, Having Both Positive and Negative Effects

**DOI:** 10.3390/jox14040077

**Published:** 2024-10-02

**Authors:** Eleonora Leti Maggio, Carlotta Zucca, Martina Grande, Raffaele Carrano, Antonio Infante, Riccardo Bei, Valeria Lucarini, Fernando De Maio, Chiara Focaccetti, Camilla Palumbo, Stefano Marini, Elisabetta Ferretti, Loredana Cifaldi, Laura Masuelli, Monica Benvenuto, Roberto Bei

**Affiliations:** 1 Department of Clinical Sciences and Translational Medicine, University of Rome “Tor Vergata”, Via Montpellier 1, 00133 Rome, Italy; eleonora.letimaggio@alumni.uniroma2.eu (E.L.M.); carlotta.zucca@students.uniroma2.eu (C.Z.); martina.grande@alumni.uniroma2.eu (M.G.); raffaele.carrano@alumni.uniroma2.eu (R.C.); demaio@med.uniroma2.it (F.D.M.); chiara.focaccetti@uniroma2.it (C.F.); camilla.palumbo@uniroma2.it (C.P.); stefano.marini@uniroma2.it (S.M.); cifaldi@med.uniroma2.it (L.C.); monica.benvenuto@uniroma2.it (M.B.); 2 Medical School, University of Rome “Tor Vergata”, Via Montpellier 1, 00133 Rome, Italy; antonio.infante@students.uniroma2.eu (A.I.); riccardo.bei@students.uniroma2.eu (R.B.); 3 Department of Experimental Medicine, University of Rome “Sapienza”, Viale Regina Elena 324, 00161 Rome, Italy; valeria.lucarini@uniroma1.it (V.L.); elisabetta.ferretti@uniroma1.it (E.F.); laura.masuelli@uniroma1.it (L.M.)

**Keywords:** polyphenols, endocrine disrupting chemicals, bisphenol-A, cadmium, phytoestrogens

## Abstract

Endocrine-disrupting chemicals (EDCs) are chemical substances that can interfere with any hormone action. They are categorized according to origin and use, such as industrial chemicals like polychlorinated biphenyls (PCBs) and polybrominated biphenyls (PBBs), plastics like bisphenol A (BPA), plasticizers like phthalates, pesticides like dichlorodiphenyltrichloroethane (DDT), fungicides like vinclozolin, and pharmaceuticals like diethylstilbestrol (DES). Natural EDCs, such as phytoestrogens, are present in the diet of both humans and animals. Polyphenols are a large group of natural compounds derived from plants and are found in beverages and food. They are grouped based on their chemical structure into flavonoids and nonflavonoids and are reported to have many beneficial effects on health, including, but not limited to, anticancer, antioxidant, and anti-inflammatory effects. Moreover, polyphenols have both pro- and antioxidant characteristics, and due to their antioxidant and anti-inflammatory potential, they presumably have a protective effect against damage induced by EDCs. However, polyphenols may act as EDCs. In this review, we report that polyphenols regulate the activity of EDCs, having both positive and negative effects. Hence, a better understanding of the associations between EDCs and polyphenols will allow the establishment of improved approaches to protect human health from EDCs.

## 1. Introduction

### 1.1. Endocrine-Disrupting Chemicals (EDCs)

Endocrine-disrupting chemicals (EDCs) are chemical substances that can interfere with any hormone action [1]. The term “endocrine disruptors” was first used by Theo Colborn and colleagues in a workshop in the early 1990s. It was formally defined by the USA Environmental Protection Agency (EPA) in 1996 as “exogenous agents that interfere with the synthesis, secretion, transport, binding, action or elimination of natural hormones in the body that are responsible for the maintenance of homeostasis, reproduction, development and/or behavior”. Initially, researchers focused their studies on the estrogenic effects of EDCs, calling them xenoestrogens [2], especially in the pre- and postnatal periods, due to all the possible risks for the fetus/newborn and for the mother.

EDCs are categorized according to their origin and use, such as industrial chemicals like polychlorinated biphenyls (PCBs) and polybrominated biphenyls (PBBs), plastics like bisphenol A (BPA), plasticizers like phthalates, pesticides like dichlorodiphenyltrichloroethane (DDT), fungicides like vinclozolin, and pharmaceuticals like diethylstilbestrol (DES). Natural EDCs, such as phytoestrogens, are present in the diet of both humans and animals. Furthermore, EDCs are divided into four main groups based on the frequency with which they occur: drugs with hormonal side effects (such as naproxen and clofibrate), natural and artificial hormones (such as phytoestrogens and contraceptive pills), industrial and household chemicals (such as phthalates, fire retardants, and solvents), and byproducts of household and industrial processes (such as polycyclic aromatic hydrocarbons (PAHs) and dioxins). Furthermore, some EDCs, such as cadmium (Cd), dioxins, phthalates, and arsenic (As), have also been recently found in tampons [3].

This classification helps in understanding the diverse nature of these chemicals and their widespread presence in various products and environments [4]. In fact, the presence of these harmful substances in many products may result in an urgent human health problem. The risk is linked to the transfer of EDCs from plastics to food, which is further increased by heat, repeated use, microwaves, and contact with acidic or alkaline solutions [5].

In recent years, attention has been given to the effects of some EDCs (called obesogens) on insulin action, considering the role of these chemical substances in the promotion of obesity and their capacity to increase the risk of type II diabetes [6,7]. It has also been demonstrated that some EDCs can interfere with bone formation [8] and immune system functions [9].

Diet is the main source of EDC intake. The consumption of canned foods, ultra-processed foods, frozen meals, soft drinks, fast food, cakes, and cookies is associated with an increase in urinary EDC levels [10,11,12,13]. Conversely, having a diet rich in minimally processed foods and/or with a high health index has been associated with low urinary levels of BPA and phthalates [14]. However, studies carried out directly on food samples to evaluate the levels of BPA have highlighted overall low levels of EDCs compared with those found in the subjects’ urine. This means that there must be another source of contamination in addition to diet [15].

#### 1.1.1. Bisphenol A (BPA)

BPA is one of the most famous EDCs, with an annual production that exceeds 3.8 million tons [16]. It is a man-made compound used in the synthesis of polycarbonates, epoxy resins, and thermal papers [17]. BPA is a white crystalline solid substance with a melting point of 156 °C. It has a hydroxyl residue directly bound to an aromatic ring, which increases its reactivity [18].

We come into contact with traces of BPA on a daily basis, given that it is present in the atmosphere, on the surface of rivers and waterways, and in dust. It has been documented that a decrease in pH in a solution is associated with increased migration of BPA from packages to food or from polycarbonate to water. BPA behaves in a very similar way to the natural estrogen 17β-estradiol (E2). At first, it was classified as a weak environmental estrogen since it has approximately 1000–10,000 times less activity towards estrogen receptor α (ERα) and ERβ than does E2. However, further research revealed that BPA bound to receptors outside of the nucleus exerts multiple effects on physiological activities in cells and tissues, even at pico- and nanomolar concentrations [19]. Furthermore, most BPA metabolites are even more effective than BPA itself [20]. Different studies have demonstrated the estrogenic effects of BPA in mice, including but not limited to an increase in prostate weight, advanced reproductive aging, earlier vaginal opening [21], alterations in the structure and function of the brain, and developmental disorders of reproductive organs [22]. In a recent study, Melzer et al. [23] demonstrated that high levels of BPA in the urine of human subjects are correlated with increased expression levels of the genes encoding ERβ and ERα, demonstrating its ability to impact the endocrine system.

Other studies have also correlated BPA with fat metabolism, revealing how this compound can interfere with the function of lipoprotein lipase and lipogenesis regulators [24], relating BPA to obesity, diabetes, and heart diseases [25,26,27].

BPA can generate reactive oxygen species (ROS) and reduce antioxidant gene expression in liver tissues, causing hepatotoxicity [28]. Furthermore, in vitro research has suggested that high doses of BPA might be harmful to nervous system cells [29]. BPA is thought to affect aryl hydrocarbon receptors (AHRs), the ERs, and possibly peroxisome proliferator-activated receptors (PPARs) and to modify immunological function [30]. Recently, it has been reported that BPA exposure affects breast cancer growth and progression in BALB–*neu*T mice by acting on tumor cells and on the tumor immune microenvironment [31]. Finally, recent studies have revealed the ability of BPA to directly damage DNA, leading to mutations and the development of neoplasms [32,33].

#### 1.1.2. Cadmium (Cd)

Another EDC is cadmium (Cd), an element found in some foods, including bivalve mollusks and crustaceans, which accumulate it by filtering the seabed, and oil seeds, such as Girasole, peanuts, flaxseed, and linseed [34]. Cd can form different compounds. Substantial variations in the release of Cd^2+^ from cadmium compounds into biological fluids, such as gastric fluid, could indicate variations in absorption and bioavailability. For example, both Cd telluride and Cd chloride (CdCl_2_) are derived from Cd, but the incorporation of CdCl_2_ in the liver and kidney can lead to increased accumulation of Cd in the tissues [35].

Cd can be stored in specific body sectors, causing various disorders and dysfunctions due to its ability to mimic the action of endogenous hormones. The long-term effects of low-dose exposure to Cd in human subjects were evaluated in the CadmiBel study, revealing tubular impairment with loss of reabsorptive capacity for nutrients, vitamins, and minerals in the kidneys [36]. The loss of calcium, which is accompanied by this absorption deficit, obviously leads to a reduction in bone tissue, resulting in a high risk of osteoporosis. Furthermore, a dose–response correlation between urinary Cd levels and an increased risk of diabetes [37] and hypertension [38] has been reported in human subjects. Moreover, low-dose Cd exposure has been correlated with an increased risk of peripheral artery disease [39,40], and high levels of Cd in the urine have been associated with a reduction of lung function [41] and age-related macular degeneration in smokers [42].

In addition, Cd is considered a carcinogen for humans because of the high incidence of lung cancer and resulting mortality in subjects occasionally exposed to sources of this element. Recently, Cd was shown to be associated not only with lung cancer but also with pancreatic [43], breast [44], endometrial [45], bladder and prostate cancer [46,47].

#### 1.1.3. Phthalates

Phthalates are a family of compounds that increase the flexibility of plastic products and are thus employed in the assembly of materials, medical devices, food packaging, and toys. Phthalate esters (PAEs) can produce a series of toxic biological effects, including immunotoxicity, endocrine and reproductive toxicity, developmental and metabolic toxicity, and genotoxicity [48]. For example, DEHP (di-(2-ethylhexyl) phthalate) is a phthalate ester present in several plastic formulations, including polyvinyl chloride (PVC), and is toxic to male reproductive organs. DEHP administration can considerably decrease the activity of testicular alkaline phosphatase (ALP), acid phosphatase (ACP), and lactate dehydrogenase (LDH), as well as markedly reduce serum testosterone levels in adult male Wistar albino rats [48]. DEHP is also able to cause liver injury [49], and exposure during pregnancy can cause hypertension in adult rat offspring due to oxidative kidney damage [50]. DEHP also has an inhibitory effect on the decidualization of endometrial stromal cells through the ER [51]. Moreover, mono-2-ethylhexyl phthalate (MEHP) is more toxic than DEHP and can induce testicular injury by increasing ROS [52].

#### 1.1.4. Atrazine (ATR)

Atrazine (ATR) is a compound that is mainly used as an herbicide and is considered an EDC because of its toxic effects on the reproductive system, nervous system, adrenal glands, and thyroid gland [53]. The toxic action of ATR has been tested in mice, where it accumulates in the liver, brain, and heart, inducing an imbalance in protein folding and the activation of the endoplasmic reticulum stress response. Overall, these effects lead to the induction of lysosomal and mitochondrial hypertrophy, mitochondrial crista rupture in cardiomyocytes, and apoptotic signaling through the action of ATF6/CHOP, which is directly correlated with the dose [54].

According to the literature, ATR disrupts several mammalian biological processes, including the formation of germ cells, the immune system, reproduction, and the nervous system. At levels at which humans are exposed, ATR lowers the sperm count and contributes to male infertility. In experimental models, ATR also caused morphological alterations comparable to those associated with apoptosis and initiated the process of mitochondria-dependent cell death [55].

#### 1.1.5. Dioxins

Dioxins are a group of compounds with high thermal and chemical stability and high resistance to degradation. They can be divided into two principal subgroups: polychlorinated dibenzo-p-dioxins (PCDDs) and polychlorinated dibenzofurans (PCDFs). The most studied dioxin is 2,3,7,8-tetrachlorodibenzo-p-dioxin (TCDD) [56]. Dioxins are formed in biomass combustions, such as forest fires, industrial and municipal waste incineration, and car exhaust. Dioxins can enter the human body through the gastrointestinal tract or contact the skin [57] and can bind to AHR, causing the expression of different genes involved in the microsomal monooxygenase system and resulting in various oxidative stresses [58].

It has been demonstrated that dioxin exposure can be related to abnormal gonadal and secondary sex trait development [59], disturbed neurological development [58], and thyroid hormone dysfunction, which is, in turn, associated with impaired psychomotor development [60]. In addition, dioxins are involved in the development of complex metabolic diseases such as diabetes or obesity [61], as well as in the formation of respiratory, gastrointestinal tract, and lymphocytic tumors [62].

#### 1.1.6. Arsenic (As)

Arsenic is a heavy metal that can be found in nature, in soil, and in groundwater [63]. It can exist in organic or inorganic form, with the latter being the most toxic to human health. Its forms include arsenious acid (As[III]), arsenic acid (As[V]), monomethylarsonic acid (MMA), dimethylarsinic acid (DMA), and trimethylarsine oxide (TMAO). Arsenic contacts the human body mostly through contaminated water, contaminated food, and soil [64].

Several studies have demonstrated an association between long- and short-term exposure to As and the development of hypertension [65]. Furthermore, this heavy metal can modify lipid metabolism, leading to a depletion of HDL cholesterol in the blood in favor of an increase in LDL cholesterol [66].

Other studies have also revealed a relationship between maternal exposure to As and the development of diabetes mellitus [67,68]. Finally, an association between exposure to As in children and the development of obesity and complex metabolic diseases was also found [69].

#### 1.1.7. Mycotoxins

Mycotoxins are natural plant-derived EDCs. These compounds can often contaminate the food supply and animal feed, which is becoming a rising concern as the global temperature increases, promoting fungal growth. In particular, zearalenone (ZEN), an estrogenic mycotoxin generated by *Fusarium* fungi, is a widespread contaminant of cereal grains and has also been found in low quantities in meat, milk, and spices. ZEN’s molecular structure resembles that of E2, which facilitates its direct interaction with nuclear ERα and ERβ. Hence, this capacity could be the reason for its greater estrogenic potency compared to other recognized endocrine disruptors [E2 > ZEN > BPA > dibutyl phthalate (DBP) > DEHP]. Given that the chemical structures of ZEN and its metabolites closely resemble those of E2, these compounds have been designated ‘mycoestrogens’ [70]. ZEN and zeranol disturb the endocrine and reproductive systems, resulting in infertility, polycystic ovarian syndrome-like phenotypes, pregnancy loss, and low birth weight [70]. It also appears that ZEN affects the morphology of primordial and primary follicles from adult sheep ovaries [71]. In vivo studies have also shown that mycoestrogens have estrogenic effects. ZEN and its metabolites bind to uterine ERα and ERβ in mice, rats, and pigs [70]. ZEN is a cause of concern due to its estrogenic properties and potential for long-term exposure. Previous research suggested that ZEN caused post-implantation mortality, and because a healthy placenta is necessary for fetal development and survival, it was suggested that ZEN might have negative effects on placental development [72]. Recently, Kinkade et al. reported alterations in sex steroid hormone concentrations in maternal circulation associated with mycoestrogen exposure, with consequences for maternal health and fetal development [73].

### 1.2. Polyphenols

**Figure 1:**
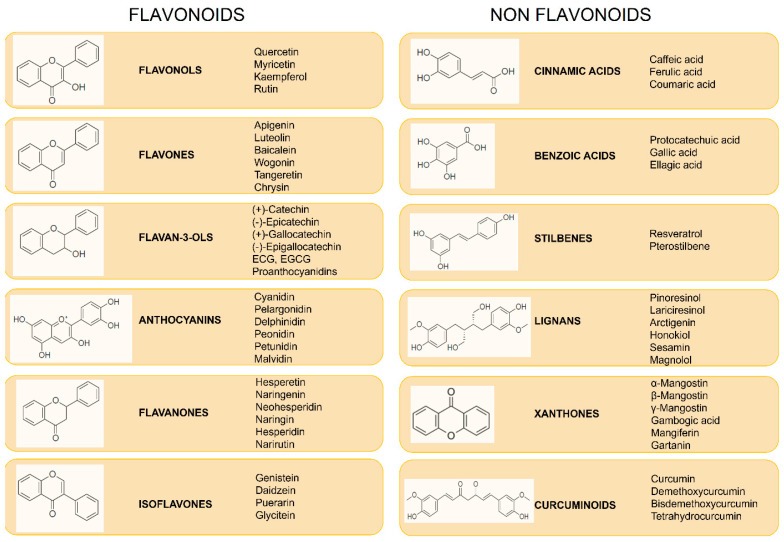
**Classification of polyphenols.** Polyphenols are classified based on their chemical structure into flavonoids and nonflavonoids. Abbreviations: ECG: epicatechin-3-*O*-gallate; EGCG: epigallocatechin-3-*O*-gallate.

Polyphenols are a large group of natural compounds derived from plants and are found in beverages and food, such as fruits, vegetables, legumes, cereals, nuts, olives, spices, tea, coffee, and wine [74]. Polyphenols are grouped based on their chemical structure into flavonoids and nonflavonoids [75,76] (Figure 1).

Flavonoids are very common in daily food intake and are derived from phenylalanine [77]. Their chemical structure consists of 15 carbon atoms and two aromatic rings (A, B) linked by a three-carbon bridge, forming a heterocyclic ring (ring C), designated C6–C3–C6 [78]. Flavonoids are divided on the basis of their different functional groups, the level of oxidation of the C’ ring, and the possible combinations of the two rings [79]. The main subclasses are flavonols, flavanones, anthocyanins, flavan-3-ols, flavones, and isoflavones [75]. In addition, the primary flavonoid core can have many substituents. The basic structure of nonflavonoids is a single aromatic ring, even though the structural skeleton of polyphenols normally contains several hydroxyl groups on aromatic rings. Examples of nonflavonoid compounds include phenolic acids, stilbenes, lignans, coumarins, curcuminoids, and xanthones [80,81]. All these plant-based compounds are reported to have many beneficial effects on health, including but not limited to anticancer, antioxidant, and anti-inflammatory effects [75,76]. Many studies indicate that, because of their antioxidant and anti-inflammatory properties, polyphenols can be used for cancer prevention and treatment, as well as for the modulation of molecular cascades involved in carcinogenesis [82,83]. In fact, polyphenols can act as antioxidants to reduce oxidative stress in the tumor context [84], and they can influence many processes involved in carcinogenesis, including the cell cycle, apoptosis, angiogenesis, and autophagy [85].

**Figure 2:**
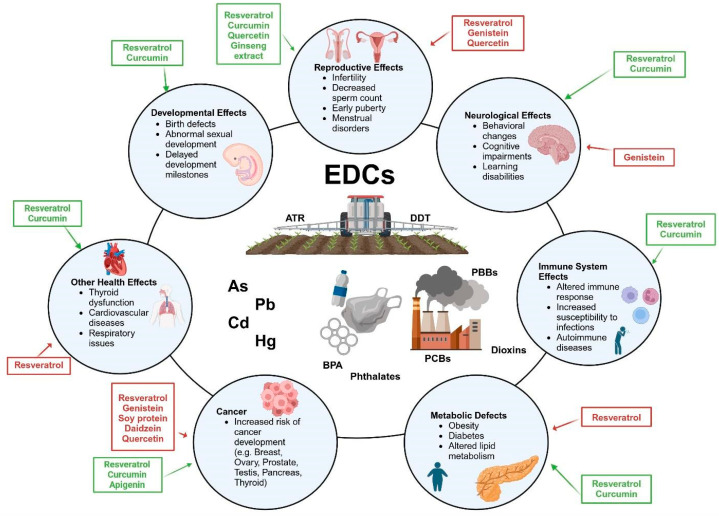
**Relationships between endocrine-disrupting chemicals (EDCs) and polyphenols.** The picture shows how EDCs can have a negative impact on human health. Polyphenols, due to their anti-inflammatory and antioxidant properties, may alleviate the harmful effects of EDCs (in green). However, because of their phytoestrogenic and pro-oxidant properties, many polyphenols may increase the harmful effects of EDCs (in red). Abbreviations: ATR: Atrazine; As: Arsenic; BPA: Bisphenol A; Cd: Cadmium; DDT: Dichlorodiphenyltrichloroethane; EDCs: Endocrine disrupting chemicals; Hg: Mercury; Pb: Lead; PBBs: Polybrominated biphenyls; PCBs: Polychlorinated biphenyls. Figure created with BioRender.com.

Moreover, polyphenols have both pro- and antioxidant characteristics [86], and due to their antioxidant and anti-inflammatory potential, they presumably have a protective effect against damage induced by EDCs [87,88]. However, those plant-derived compounds may act as EDCs or become such after exposure to contaminants. Different studies have shown that these compounds are also capable of negatively interacting with hormone modulation pathways [88,89]. Estrogens are a group of steroid hormones that are important in females for sexual and reproductive development and influence many other important functions in the human body, such as cell proliferation and death, lipid metabolism, glucose metabolism, and immune and cardiovascular regulation [90]. Moreover, polyphenols, defined as phytoestrogens, can also interact specifically with ERα and ERβ since they are structurally very similar to E2, the strongest estrogen [91]. E2 operates via ERα and ERβ receptors throughout the body; therefore, the biological effects of polyphenols on the human body may extend beyond the modulation of oxidative stress [92]. These phytoestrogens act as endogenous estrogens and consequently may act as EDCs of estrogen metabolism [93]. This possibility needs to be considered because polyphenols are present at much higher concentrations in dietary supplements than are normally present in the human diet, which could lead to the known damaging effects of EDCs [94] (Figure 2).

## 2. Endocrine-Disrupting Chemicals and Polyphenols: A Disjointed Action

Polyphenols are known to have positive effects on human health due to their antioxidant and anti-inflammatory properties, which makes them very important in the medical field, especially for cancer treatment. The following paragraph reviews the studies that highlight these effects, emphasizing how these compounds can counteract EDCs.

### 2.1. Effects on Female Reproductive System

Different polyphenols have been found able to protect the female reproductive system against the toxicity induced by EDCs.

Abady et al. reported the negative impact of BPA and the protective roles of melatonin (MT) and resveratrol (RES) in endometrial organoids, suggesting possible therapeutic approaches for reproductive health. Their results showed that MT and RES may have a protective effect against BPA-induced morphological alterations, oxidative stress, and apoptosis in porcine endometrial organoids. Furthermore, BPA-induced alterations in epithelial markers were reduced by MT and RES. The protective effects of these two compounds were also highlighted by their ability to modulate Wnt/β-catenin signaling [95]. In another study on the female reproductive system, Fouad et al. investigated the potential benefits of mesenchymal stem cells (MSCs) and RES in an experimental model of BPA-induced uterine injury in rats [96]. MSCs and RES worked together to restore normal gonadal hormone synthesis, reduce oxidative stress, decrease apoptosis, generate antifibrotic effects, and ameliorate histological damage. The combined therapy produced better therapeutic results than the single treatment [96].

Another study was conducted by Piras et al. to investigate whether treatment with RES may protect against Cd-induced toxicity during ovine oocyte maturation and fertilization. The study indicated that RES repaired the impaired oocyte meiotic competence caused by Cd exposure and maintained the oocyte’s capacity to be fertilized, preventing polyspermic fertilization. Furthermore, they demonstrated that RES mitigated Cd-induced alterations in oocyte cytoplasmic maturation by reducing ROS accumulation, preventing mitochondrial dysfunction, maintaining correct meiotic spindle and cortical F-actin assembly as well as normal cortical granule distribution, and upregulating Sirtuin 1 (SIRT1), superoxide dismutase 1 (SOD1), and glutathione peroxidase 1 (GPx1) genes [97]. Wang et al. studied the effects of RES on Cd-induced placental damage in pregnant CD-1 mice. The results showed that Cd exposure reduced fetal weight and crown-rump length, whereas RES improved these parameters. RES reduced Cd-induced placental toxicity by regulating DNA methyltransferase (DNMT) expression and PI3K/Akt pathway activation and by alleviating endoplasmic reticulum stress in mouse placentas [98]. Another study by Liu et al. uncovered the toxic effect of BPAF (bisphenol AF, a BPA replacement) using an in vitro culture model of caprine endometrial epithelial cells (EECs) and evaluated CUR pretreatment as an attempt to mitigate BPAF toxicity. This study showed that BPAF has significant effects on EECs, including decreased cell viability and mitochondrial membrane potential (△ψm), increased intracellular ROS, and promotion of cell apoptosis via the upregulation of Bax and cytochrome c expression and the downregulation of B-cell leukemia/lymphoma 2 protein (Bcl-2). However, CUR pretreatment considerably reduced BPAF-induced toxicity in EECs. CUR pretreatment prevented the activation of the MAPK signaling pathway and Nrf2 expression caused by BPAF [99]. Another interesting study focused on assessing the impact of zearalenone and matairesinol (MAT), a polyphenol, on the morphology of in vitro-cultured ovarian preantral follicles. ZEN affected the morphology of primordial and primary follicles. The plant lignan MAT alone did not preserve the morphology of ovarian follicles, but its combination with ZEN mitigated the negative effects observed when the mycotoxin was used alone [71]. Wang et al. discovered that, while BPA exposure during lactation promoted the proliferation of abnormal mammary gland cells later in life, the co-exposure to genistein increased the normal proliferation and differentiation of mammary gland structures earlier in life and significantly lowered abnormal proliferation later in life [100]. Finally, concerning the actions of RES against the effects of BPA on the female reproductive system, it is interesting to highlight the findings of Jiao et al., who reported that BPA disrupted the mouse estrus cycle by lowering progesterone and estradiol levels. BPA increases oxidative stress, autophagy, and apoptosis in ovaries and granulosa cells. However, RES improved estrous dysfunction and estradiol secretion in BPA-exposed ovarian tissues by reducing aberrant ROS buildup, autophagy, and apoptosis [101]. Gao et al. demonstrated that genistein ameliorated BPA-induced oxidative stress in adult ovaries of laying hens by acting on the ERα and Keap1-Nrf2 signaling pathways [102].

Ginseng extract can reduce the reproductive toxicity effects of phthalates (such as DEHP) and BPA in pregnant rats by restoring abnormal steroid hormone levels to normal levels and modulating the mRNA transcripts of steroidogenic enzymes, either directly or via the Akt/PTEN pathway [103].

Moreover, it has been shown that RES can counteract the proliferative effects of several EDCs, including BPA, octylphenol (OP), and methoxychlor (MXC) in BG-1 ovarian cancer cells. Indeed, ovarian cancer cells treated with the EDCs alone had increased proliferation, whereas those treated with both EDCs and RES had a decreased proliferation [104]. Using the same cell line, Kang et al. examined the pro-survival effects of BPA as well as the growth-inhibitory effect of RES. The BG-1 cell line is composed of estrogen-dependent cancer cells that substantially express the ER isoforms ERα and ERβ and overexpress insulin-like growth factor binding proteins when exposed to E2 or BPA. They reported that RES suppressed the growth of BG-1 cancer cells by disrupting the interaction between the ERα and IGF-1R pathways, hence limiting cell cycle progression [105]. Bulzomi et al. found that naringenin increased apoptosis by activating the p38 pathway in human estrogen-dependent breast cancer cell lines (MCF-7 and T47D) and inhibited cell proliferation caused by BPA exposure by blocking its interaction with the ERα receptor and by preventing BPA-induced Akt activation. Thus, naringenin may have the ability to counteract BPA’s cancer-promoting effects [106].

### 2.2. Effects on Male Reproductive System

Several studies have highlighted the protective role of different polyphenols against the toxicity induced by EDCs on the male reproductive system.

Mitra et al. reported that long-term exposure to CdCl_2_ and lead acetate [Pb(CH_3_COO)_2_] caused acute reproductive damage and the onset of testicular germ cell neoplasia in situ (GCNIS) in mice. RES consumption suppressed the metal-induced perturbation of spermatogenesis, testicular morphology, and the upregulation of Akt cascade proteins along with GCNIS markers [107]. Moreover, Bordbar et al. investigated the effects of low and high doses of BPA on testicular structure and sperm quality in male Sprague–Dawley rats, describing how RES treatment ameliorated the effects of BPA. When BPA was administered, there was a significant decrease in sperm parameters as well as a reduction in testicular diameter, which were reversed when RES was used as a cotreatment, leading to increased levels of gonadotropin hormone and testosterone [108]. Similarly, quercetin has shown protective effects against BPA-induced testicular damage in rats [109]. Another study on this topic was carried out to investigate the therapeutic effects of CUR and quercetin in combination on ATR-induced testicular damage in rats. According to their findings, ATR challenge decreased luteinizing hormone, follicle-stimulating hormone, testosterone, and myeloperoxidase enzyme activity. These effects were mitigated by the cotreatment with CUR and quercetin, which was superior to the effects of quercetin alone. Finally, they demonstrated that CUR could enhance the protective effects of quercetin against ATR-induced testicular injury by increasing reproductive hormone levels, restoring testicular biochemical parameters, and improving testicular histological features [110]. Another study investigated the effects of nutraceuticals such as a flavonoid-rich extract of bergamot juice (BJe), either alone or in combination with CUR and RES, on adult male C57BL/6J mice with CdCl_2_-induced testicular dysfunction. Its findings suggest that BJe decreases Cd-induced testicular damage via an anti-inflammatory and antiapoptotic mechanism. Furthermore, the results of this study show that a combination of both CUR and RES can strengthen the protective impact [111]. DEHP administration can also cause considerable decreases in the activity of testicular alkaline phosphatase (ALP), acid phosphatase (ACP), and lactate dehydrogenase (LDH), as well as a marked reduction in serum testosterone levels in adult male Wistar albino rats. The pretreatment with RES and CUR counteracted these effects [48]. Reversal of DEHP-induced testicular injury was also observed after gallic acid treatment in adult male NMRI mice [112]. RES also had a protective effect against damage to the ductus epididymis and deferens provoked in rats by di-n-butyl phthalate (DBP) [113]. Similarly, Berköz et al. demonstrated the preventive role of the natural aromatase inhibitors RES and apigenin against BPA-induced testicular failure [114]. In addition, the efficacy of CUR, either alone or in combination with piperine, in suppressing the effect of BPA was investigated in the prostate of adult male gerbils (*Meriones unguiculatus*). BPA induced prostatic inflammation and morphological abnormalities in the ventral and dorsolateral prostate lobes, along with an increase in the number of androgen receptor (AR) (+) cells and nuclear atypia, primarily in the ventral lobe. CUR and piperine helped to reduce these pathological changes [115]. According to Samova et al., quercetin may mitigate the negative impact of BPA on steroidogenesis. This action may be due to quercetin’s tendency to bind to key enzymes involved in the process, including 17β-hydroxysteroid dehydrogenase and 3β-hydroxysteroid dehydrogenase. Indeed, quercetin boosted the activity of these enzymes, which was accompanied by a rise in total lipids and testosterone levels in animals treated with a quercetin-BPA mixture [116].

Finally, *Eruca sativa* aqueous extract (ESAE) was found able to protect against the toxic effects of BPA on human spermatozoa. In particular, it has been reported that ESAE at low concentrations can restore membrane potential and sperm motility after BPA treatment [117].

### 2.3. Effects on Gastrointestinal System

Several studies have highlighted the protective role of different polyphenols against the toxicity induced by EDCs on the gastrointestinal system.

For example, quercetin administered in the diet of BPA-exposed mice prevented liver enlargement, decreased ALT, AST, and ALP activity, and reduced blood creatinine levels. Polyphenols’ hepatoprotective impact against BPA is likely due to their antioxidant and anti-inflammatory properties [118]. Liao et al. reported that resveratrol butyrate ester (RBE) administered to offspring rats effectively reduced BPA-induced oxidative damage in the liver in addition to reducing alanine aminotransferase (ALT) and aspartate aminotransferase (AST) activities. Furthermore, RBE effectively increased mRNA expression and activities of antioxidant enzymes, improving liver function overall and reducing oxidative damage. In addition, when maternal rats ingested BPA, abnormalities in the gut microbiota distribution of their offspring occurred, leading to a reduction in intestinal barrier function and the development of inflammatory responses and oxidative damage in offspring livers [119]. Silymarin, another polyphenol with hepatoprotective effects, is widely used in dietary supplements [120]. A study on mice found that silymarin counteracted the adverse effects of BPA by reducing inflammation in liver tissue. Silymarin inhibited the expression of interleukin (IL)-6 and tumor necrosis factor (TNF)-α genes, avoiding BPA-induced alterations in liver tissue ultrastructure [120]. In addition, oral treatment of BPA-exposed rats with CUR or taurine reduced lipid peroxidation and increased the level of GPx and GST, CAT, and SOD activities. Furthermore, the treatment decreased inflammatory cell infiltration and prevented necrosis in the liver of the rats. Thus, CUR and taurine can partially protect rats from BPA-induced hepatotoxicity [121]. Similarly, lycopene treatment reduced BPA’s cytotoxic effect on hepatic tissue by enhancing liver function indicators, regulating the oxidative-antioxidant state, and reducing DNA damage. Lycopene’s beneficial antioxidant effect was confirmed by the higher antioxidant activity of SOD, GPx, and CYP, as well as by the lower levels of malondialdehyde (MDA) in rats co-treated with BPA and lycopene [122].

CUR also prevents BPA-induced hepatic steatosis by limiting intestinal cholesterol absorption and hepatic cholesterol synthesis, lowering liver cholesterol accumulation, and ultimately improving liver lipid biosynthesis and fat accumulation. These findings support the use of CUR as a possible supplement to protect against BPA-induced hepatic steatosis [123]. Moreover, Elswefy et al. reported that CUR, n-acetyl cysteine (NAC), and propolis extracts exert hepatoprotective effects on BPA-induced fibrosis by reducing oxidative stress, inflammation, apoptosis, and extracellular matrix turnover [124]. The combination of naringenin with vitamins C and E enhanced Cd detoxication in rats’ liver tissue by suppressing oxidative stress, enhancing antioxidant status, and reducing histopathological alterations [125].

Further findings indicate that DEHP is also able to cause liver injury. Indeed, DEHP was reported to induce liver dysfunction in C57BL/6J mice, which was alleviated by green tea polyphenols through miRNA–mRNA-protein molecular modifications [49]. In studies performed on male mice treated with DEHP, it was found a notable decrease in the activity of critical liver enzymes in the blood (ALT and AST) as well as a significant rise in liver expression of the CYP3A4 gene, which is one of the essential enzymes in drug metabolism. The administration of EGCG to DEHP-treated mice almost entirely restored the values to those found in the control group [49].

Çetin et al. investigated the cellular alterations caused by BPA exposure in rat salivary gland cells, as well as the protective role of RES and apigenin. Their results showed that RES and apigenin decreased tissue oxidative stress and increased tissue antioxidant levels. BPA also produced cytopathological alterations and apoptosis in salivary gland cells, as well as edema, nuclear pleomorphism, and the formation of pyknotic nuclei, whereas both RES and apigenin protected the cells against BPA-induced damage, with RES providing greater protection than apigenin [126].

Remarkably, RES appears to play a key role in reversing the effects of Cd on colorectal cancer (CRC) cells. In this regard, it has been shown that, while Cd induces the epithelial–mesenchymal transition (EMT) in CRC cell lines, RES could prevent Cd-induced migration and invasion of CRC cells in vitro by controlling the m6A alteration of zinc finger E-box binding homeobox 1 (ZEB1) and the expression of EMT-related markers [127].

Finally, according to a recent study, CUR ameliorated the toxic effects of BPA on the glandular portions of the stomach of rats thanks to its antifibrotic and antiapoptotic properties [128].

### 2.4. Effects on the Urinary System

Zhang et al. demonstrated the effects of RES against Cd-induced nephrotoxicity in one-year-old male Hy-Line Variety White chickens. Their findings indicate that RES can inhibit Cd-induced nephrotoxicity and destruction of kidney structure. Notably, RES increased the activity of antioxidant enzymes and decreased Cd-induced severe oxidative stress. RES supplementation enhanced phase II detoxification and balanced redox reactions by activating the nuclear xenobiotic receptor (NXR) and nuclear factor erythroid 2-related factor 2 (Nrf2) signaling pathways. Furthermore, RES reversed Cd-induced alterations in the mitochondrial ultrastructure, alleviated mitochondrial dysfunction, and restored mitochondrial biogenesis by inhibiting excessive mitochondrial fission and mitophagy [129]. Renugadevi and Prabu revealed that quercetin protected rats’ kidney tissue from oxidative damage caused by Cd. This polyphenol was able to restore the levels of the enzymatic antioxidants catalase (CAT), SOD, GPx, and GST, as well as those of non-enzymatic antioxidants (vitamins C and E and reduced glutathione) in the kidney, reducing lipid peroxidation and some other biochemical parameters such as urea, uric acid, and creatinine levels [130].

The nephroprotective effect of luteolin on BPA-induced toxicity was studied in rats. Luteolin decreased BPA-induced renal problems, reducing blood urea nitrogen, serum creatinine, and serum uric acid levels [131].

Ren et al. demonstrated that the triazine pesticide ATR has cytotoxic effects on mouse TCMK-1 renal cells due to oxidative stress-mediated cellular pyroptosis and DNA damage. CUR significantly reduced ATR-induced TCMK-1 cell pyroptosis and cell cycle arrest by decreasing oxidative stress [132].

### 2.5. Effects on the Brain and Nervous System

It has been reported that quercetin has a neuroprotective effect against Cd exposure [133]. In addition, Tiwari et al. investigated the neuroprotective role of CUR in decreasing the negative effects of BPA on rat hippocampal neurogenesis and cognitive functions. CUR has been identified as an effective neuroprotective compound in models of Parkinson’s disease, Alzheimer’s disease (AD), and amyotrophic lateral sclerosis (ALS). For instance, CUR increased adult hippocampal neurogenesis and corrected learning and memory deficits induced by BPA in AD models via the activation of the Wnt/β-catenin pathway [134]. Tandon et al. reported that CUR protected BPA-exposed rats both in vitro and in vivo. These findings further emphasize the role of canonical Notch signaling in CUR-mediated neuroprotection and highlight the usefulness of CUR as a possible therapeutic drug against BPA neurotoxicity [135].

Finally, Li et al. demonstrated that isoflavones could increase brain-expressed X-linked 2 (BEX2) levels and activate BEX2-dependent autophagy, preventing neuronal cell death caused by ATR. Indeed, ATR reduces tyrosine hydroxylase and BEX2 expression while increasing dopaminergic neuronal cell death. These findings imply that isoflavones and their neuroprotective properties are promising therapeutic tools for the prevention and/or treatment of neurodegenerative illnesses such as Parkinson’s disease [136].

### 2.6. Effects on Endocrine System

Several studies have highlighted the protective role of different polyphenols against the toxicity induced by EDCs on the endocrine system.

Rameshrad et al. investigated BPA-induced metabolic syndrome and the preventive effects of grape seed extract and RES in male albino Wistar rats. BPA negatively affected blood pressure, hepatic expression of ABCG5 and ABCG8, and the lipid profile. Furthermore, chronic BPA exposure reduced paraoxonase-1 (PON1) serum concentrations while increasing leptin, adiponectin, and body fat index levels. It disrupted insulin signaling by increasing fasting blood sugar and the serum insulin concentration and decreasing the hepatic phosphorylated-Akt/Akt and phosphorylated-phosphatidylinositol-3 kinase (PI3K)/PI3K ratios. In this context, the protective benefits of grape seed extract and RES may be linked to their impact on hepatic ABCG8 expression, improved insulin signaling, and antioxidant capabilities [137]. Using the same model, another study investigated the interactions of BPA and RES by evaluating the expression of several proteins involved in the proliferative pathway. BPA treatment significantly increased the serum levels of α-amylase, α-glucosidase, glucose 6 phosphatase, insulin, hemoglobin A1c (HbA1c), 3-hydroxy-3-methylglutaryl (HMG)-CoA reductase, free fatty acids (FFAs), triglycerides (TGs), dipeptidyl peptidase 4 (DPP-4), MDA, and proinflammatory cytokines such as TNF-α and IL-6. Conversely, the levels of antioxidant enzymes such as CAT, GPx, SOD, glucose transporter type 4 (GLUT4), and HDL cholesterol were significantly decreased. When treated with RES at a later time, the rats that had been treated with BPA showed fewer alterations, suggesting that RES might have a protective effect [138]. In another study, RBE was administered to rats exposed to BPA throughout the perinatal period to investigate how RBE supplementation could affect obesity-related markers and the intestinal microbiota in their female offspring. The findings of this study demonstrated that RBE supplementation can reduce BPA-induced weight gain and body fat accumulation and increase the concentration of blood lipid-related markers. Furthermore, the authors demonstrated that RBE supplementation can influence the intestinal concentration of acetate in female offspring rats [139]. Geng et al. reported that BPA increased glucose consumption, insulin signaling, oxidative stress, inflammatory cytokines, as well as MAPKs and nuclear factor kappa B (NF-κB) pathway activation in hepatic HepG2 cells, whereas CUR had a preventive effect. These findings revealed that BPA administration can induce insulin resistance in hepatic cells, which was mediated by JNK/p38 MAPK activation, and that CUR can act as a preventive agent against BPA-induced insulin resistance. Previous research has shown that the etiology of insulin resistance is intimately related to inflammation and oxidative stress, so treatments for insulin resistance may work by decreasing inflammation and stress. In fact, CUR significantly reduces TNF-α and IL-6 release, as well as MDA and cyclooxygenase-2 (COX2) levels, indicating its ability to inhibit inflammation and oxidative stress [140,141]. It has been reported that BPA produced persistent inflammation in the lungs of rats and increased the levels of IL-1, IL-6, Bax, and caspase-3 expression. Lycopene administration was able to reduce the harmful effects of BPA and restore all oxidant/antioxidant markers to near-normal levels [142]. Moreover, RES stimulates the early maturation of osteoblastic MC3T3-E1 cells and significantly reduces CdCl_2_-induced suppression of osteogenic differentiation [143].

### 2.7. Effects on Cardiovascular System

Hsu et al. reported that maternal RES treatment reduces hypertension in adult male offspring caused by combined BPA and high-fat (HF) exposure by targeting the asymmetric dimethylarginine-nitric oxide (ADMA-NO) pathway, oxidative stress, and AHR signaling in the kidney. According to their findings, maternal BPA exposure exacerbates hypertension in adult male offspring induced by dams fed an HF diet, whereas RES alleviates hypertension caused by BPA or HF diet plus BPA. The HF diet and BPA exposure had a synergistic effect on the generation of oxidative damage in offspring kidneys, which was prevented by RES therapy. In particular, hypertension induced by HF diet plus BPA was linked to decreased NO bioavailability, increased oxidative stress, and activation of the AHR signaling pathway. RES was able to restore NO bioavailability, decrease oxidative stress, and inhibit AHR signaling, thereby preventing HF + BPA-induced hypertension in adult male offspring [144]. Notably, DEHP exposure during pregnancy caused hypertension in adult rat offspring due to kidney oxidative damage, but RBE supplementation during gestation and lactation protected the offspring of DEHP-exposed pregnant rats from developing hypertension [50].

In an interesting study, Apaydin et al. administered olive oil, CUR, taurine, BPA, CUR plus BPA, and taurine plus BPA to adult male albino rats via gavage for four weeks. They reported substantial alterations in MDA levels and antioxidant enzymes activity (GPx, GST, SOD, and CAT) in rats exposed to BPA, as well as histopathological abnormalities, but CUR and taurine dramatically reduced the BPA-induced cardiac damage [145].

Liang et al. reported that ATR creates cardiac endoplasmic reticulum stress in mice via ATF6/CHOP signaling, resulting in apoptosis and heart injury. Nonetheless, CUR had the potential to treat heart damage by reducing endoplasmic reticulum stress and cardiac apoptosis. These findings shed light on the role of CUR as a protective agent against ATR-induced damage in animals [54]. Wang et al. investigated how apigenin affects inflammation in DEHP-treated HUVEC cells. Their findings reveal that DEHP stimulates the expression of IL-8 and ICAM-1 by activating the ERK1/2 and p38/MAPK pathways. On the other hand, apigenin inhibited the expression of IL-6, IL-8, and ICAM-1 in DEHP-treated cell cultures [146]. Mohsenzadeh et al. reported that in rat aortic and HUVEC cells, EGCG increased vascular responsiveness and reduced BPA-induced elevations in MDA, cleaved caspase-3, LC3A/B, Bax/Bcl2 ratio, and VCAM-1. As a result, EGCG may be effective in lowering BPA-induced vascular dysfunction and slowing the progression of atherosclerosis [147]. To study the effects of RES on perinatal BPA exposure-induced atherosclerotic lesions, Sirasanagandla et al. used an apolipoprotein E-deficient (ApoE^−/−^) mouse model. Their results reveal that maternal RES supplementation dramatically reduces BPA-induced atherosclerotic alterations in adult offspring. This outcome might be due to RES acting as an autophagy regulator and/or epigenetic modifier [148].

In conclusion, many polyphenols, such as RES, CUR, isoflavones, ESAE, apigenin, and several others, have beneficial effects on human health, some of which appear to involve their protective effects against EDCs action (Table 1).

## 3. Endocrine Disrupting Chemicals and Polyphenols: A Synergic Action

Polyphenols also have the capacity to enhance the detrimental effects of EDCs by functioning as phytoestrogens via the tissue-specific modification ofER and AR [150].

Phytoestrogens have a wide range of properties that interfere with the hormonal regulation of homeostasis. The main mechanisms through which phytoestrogens can exert possibly harmful effects: (i) by acting as ligands at the hormone’s binding sites and mimicking the effects of the natural, endogenous ligand; (ii) by blocking the interaction of the endogenous hormone with its physiological binding sites and antagonizing its effect; (iii) by reacting with a given hormone; (iv) by altering the natural patterns of hormone production and degradation; and (v) by disrupting cellular hormone receptor expression [151,152]. Furthermore, it has been proven that lifetime exposure to estrogen-like compounds, particularly during critical developmental phases, is linked to the development of cancer and a variety of reproductive defects [153]. ER signaling pathways commonly involve growth factor receptors and G protein-coupled receptors (GPCRs). GPER (GPER1, or GPR30), a seven-transmembrane-domain receptor from the GPCR superfamily, is one of the first identified receptors that mediates estrogen-dependent kinase activation and transcriptional responses [154]. Several phytoestrogens, such as flavones (e.g., quercetin), isoflavones (e.g., genistein), lignans, coumestans, saponins, and stilbenes, have been shown to activate GPCRs [155]. For example, genistein and quercetin can increase c-fos expression in ER(+) MCF7 and ER(−) SKBR3 breast cancer cells, i.e., in an ER-independent manner, via GPER [154,155,156]. Many studies have demonstrated the hormonal activities of isoflavones, while studies on genotoxic activity are still limited. The available studies focused on genistein and daidzein [157,158]. Genistein can be classified as a phytoestrogen of soy-derived food and acts as a partial agonist of ERα. Intake of genistein may affect leptin hormone, C-reactive protein, tyrosine kinase activity, and thyroid function [159]. In the most recent study on male rats, consumption of relevant doses of soy isoflavones during the peripubertal period induced subclinical hypothyroidism, with alterations in the regulation of the hypothalamic-pituitary-thyroid axis, modulation of thyroid hormone synthesis, and peripheral alterations in thyroid hormone target organs [160]. In several experimental models, genistein was found to produce tumors in mice [157,158]. Recent research has shown that long-term intake of modest levels of genistein leads to hormone-independent growth of MCF-7 cancer cells with increased HER2 levels. In fact, Hu et al. reported that low doses of genistein had estrogen-like effects and suppressed HER2 expression after short-term exposure in ER(+) breast cancer cells. Long-term exposure, however, increased HER2 expression, leading to endocrine resistance [161]. Balázs et al. studied the cytotoxic and estrogenic effects of genistein, ZEN, its metabolites, and alternariol (AOH), a mycotoxin produced by Alternaria species, to evaluate their individual and combined impacts on a human cervical cancer cell line (HeLa). Their results suggest that genistein, in addition to ZEN metabolites and AOH, can increase ZEN-induced toxicity. Furthermore, the compounds exerted synergistic or additive effects on cytotoxicity and/or estrogenicity [162].

Despite being associated primarily with estrogens, genistein also has antiandrogenic properties. A clinical trial revealed that patients with a high risk of developing advanced prostate cancer had lower AR levels in tumor cells after consuming a rich soy protein isolate diet, although ERβ expression remained unaffected [163]. In addition, Terzioglu-Usak et al. demonstrated that genistein appears to induce dose-dependent responses. At concentrations lower than 10 μM, it increased the proliferation of PC3 cells, whereas at concentrations greater than 10 μM, it had cytotoxic effects, resulting in decreased cell viability and migration [164]. Moreover, Pihlajamaa et al. reported that genistein exhibits a tissue-specific AR response, making it a possible selective AR modulator (SARM) and not just an AR agonist. Administering genistein daily for five days to transgenic male mice induced an antiandrogenic response in the testis, prostate, and brain. However, in castrated males, the treatment activated the AR only in the prostate and the brain. These findings indicate that genistein could behave as a partial agonist/antagonist in the prostate, depending on the level of circulating androgens [165]. The use of appropriate doses when investigating these compounds is crucial because of their many cellular targets, which can greatly impact therapeutic outcomes.

Another naturally occurring compound found exclusively in soybeans and other legumes is daidzein (7-hydroxy-3-(4-hydroxyphenyl)-4H-chromen-4-one). Eastern people, especially Japanese and Korean people, have an intestinal flora that can convert daidzein into equol, the most physiologically active isoflavone [166,167,168]. This compound exists in two enantiomers: S-equol and R-equol. S-equol has a greater binding affinity for ERβ, whereas R-equol preferentially binds ERα. Both have a better affinity for ERs than their precursor, daidzein [169]. Compared with S-equol alone, 1 μM R-equol significantly increased proliferation in MCF-7 cells (ERα(+), ERβ(−)) [170]. In pregnant rats, the phytoestrogen daidzein was shown to pass rapidly from mother to fetus. Following the intravenous injection of daidzein to the mother, its concentration in the placental tissue and fetal liver was 1/10 and 1/30 of the maternal liver’s peak concentration, respectively [171].

Shahzad et al. observed that low doses of quercetin alter uterine morphology but not proliferation; however, higher concentrations of quercetin induced considerable stromal and glandular proliferation, which may predispose the uterus to neoplastic growth [172]. In addition to quercetin, many other plant chemicals, including genistein at high doses, have been shown to increase uterine proliferation, which may cause neoplastic alterations [173,174]. Also, consuming soy raises phytoestrogen concentrations in amniotic fluid in both male and female infants [175].

Another study investigated how rooibos (*Aspalathus linearis* Brum. F) and quercetin (a flavonol) can influence the response of ovarian cells to follicle-stimulating hormone (FSH). The authors tested the effects of rooibos extract and quercetin on porcine ovarian granulosa cells cultured with and without FSH. This treatment resulted in a lower accumulation of proliferation markers while promoting an increase in apoptosis markers and the release of testosterone and estradiol. Notably, rooibos increased progesterone output, whereas quercetin decreased it. FSH administration increased the accumulation of proliferation markers, decreased the accumulation of apoptotic markers, increased progesterone and testosterone release, and had a biphasic effect on estradiol output. In conclusion, the combination of rooibos and quercetin reduced or prevented the primary effects of FSH, suggesting that rooibos and quercetin both have a direct influence on basic ovarian activities such as proliferation, apoptosis, steroidogenesis, and FSH responsiveness. Given that quercetin is the main component of rooibos, it may be the molecule responsible for rooibos’ primary actions on the ovary; therefore, the potential anti-reproductive effects should be considered in both animal and human nutrition [176].

Similarly, another investigation reported that RES and other chemicals with estrogenic activity, such as ATR, diethylstilbestrol (a synthetic estrogen), para-nonylphenol (para-nonylphenol), E2, can potentially have harmful effects on pregnancy success and fetal health. This study reported that these chemicals at concentrations ranging from 1 μM to 1 mM decreased cell viability and β-hCG secretion in trophoblast-derived choriocarcinoma cells [177]. In another study, Botelho et al. reported that the administration of RES and vitamin C is not able to correct DEHP-induced modifications in the reproductive endpoints of newborn male rats exposed in utero. Instead, the two antioxidants worsened both oxidative stress and damage [178]. Because estrogens have been shown to inhibit the formation and activity of Leydig cells, there have been reports regarding the impact of RES on the function of steroidogenic cells. Li et al. demonstrated that RES could be a possible endocrine disruptor of Leydig cell function by inhibiting LH-stimulated androgen synthesis in rat Leydig cells. Further research revealed that RES mostly reduced 3β-HSD activity in a competitive way. This finding is significant, as RES demonstrated similar suppression of human testosterone biosynthetic enzyme activity [179]. Gehm et al. (1997) tested RES at approximately 3–10 μM, the concentration required for its other biological effects. They reported that it inhibited the binding of labeled estradiol to the ER and activated the transcription of estrogen-responsive reporter genes when it was transfected into human breast cancer cells. In some cell types (e.g., MCF-7 cells), RES works as a superagonist, producing a greater transcriptional response compared with estradiol. Moreover, RES enhanced the expression of native estrogen-regulated genes and accelerated the growth of estrogen-dependent T47D breast cancer cells [180]. In addition, RES promoted breast tumor growth and metastasis in immunocompromised mice implanted with low-metastatic ERα(−), ERβ(+) MDA-MB-231, and highly metastatic ER(−) MDA-MB-435 cancer cell lines. Taken together, these findings suggest that small amounts of RES may promote breast cancer, emphasizing the importance of better understanding the concentration-dependent effects of this compound, particularly in breast cancer, before it can be investigated in the clinic or used as a dietary supplement for breast cancer patients. Furthermore, the activities of the migration/invasion regulator Rac, along with its downstream effector PAK1, were enhanced by RES treatment [181].

In addition to ERs, EDCs have several targets that play a role in the endocrine system, such as human aromatase and cytochrome P450, which catalyze the conversion of androgens into estrogens. RES is a potent aromatase inhibitor and, thus, can exert strong endocrine-disrupting activity by decreasing the concentration of estrogen sex hormones [182].

RES can also increase the expression of some thyroid-specific genes in human thyroid anaplastic carcinoma cell lines, particularly the sodium/iodide symporter (NIS) gene [183]. Another study recently revealed that RES transiently enhances iodide influx in FRTL-5 rat thyroid cells. This increase occurred after a brief treatment [184]. Moreover, Giuliani et al. evaluated the effects of RES on iodide uptake and sodium/iodide symporter expression in thyroid cells after extended periods of treatment. Both in vitro and in vivo experiments, employing the rat thyroid FRTL-5 cell line and Sprague–Dawley rats, revealed that RES lowered RNA and protein levels of the sodium/iodide symporter and reduced cellular iodide uptake after 48 h of treatment. This study demonstrated that RES inhibits the expression and activity of the sodium/iodide symporter gene in the thyroid when it is used as a long-term drug [185]. Similarly, the effects of RES on the expression of additional thyroid-specific genes, such as thyroglobulin, thyroid peroxidase, the Thyroid-stimulating hormone (TSH) receptor, Nkx2-1 (also known as TTF-1), Foxe1 (also known as TTF-2) and Pax-8, were evaluated in the FRTL-5 rat thyroid cell line. Compared with those in control cells, the levels of these genes in cells treated with RES were lower. The effects of RES were subsequently investigated in vivo. Serum TSH and thyroid hormone levels were within normal ranges, with considerably increased TSH in RES-treated rats compared with control rats. Histological and immunohistochemical tests indicated that rats treated with RES presented increased thyroid proliferative activity. These findings suggest that RES may act as a thyroid disruptor, highlighting the need for caution when it is used as a food supplement or for medical use [186].

In summary, research has shown that polyphenols can also act as EDCs and might have harmful effects on human health (Table 2).

## 4. Conclusions and Future Perspectives

In recent decades, polyphenols have gained attention in medical research for their beneficial health properties, including anti-inflammatory and antioxidant effects. Several studies have explored their potential in the clinical setting, focusing on their beneficial effects on human health, associated with the reduction of oxidative stress, the improvement of metabolic health, and the modulation of inflammatory pathways. Despite these promising findings, recent research also indicates that many polyphenols are phytoestrogens and thus can act as EDCs under certain conditions, highlighting the need to understand both their positive and negative effects.

There is a substantial gap in clinical research on the combined effects of EDCs and polyphenols despite the growing evidence from in vitro and animal studies suggesting that polyphenols may mitigate or, in some cases, exacerbate the harmful impacts of EDCs. While polyphenols have been demonstrated to exert protective effects by improving antioxidant defenses and reducing inflammation, and even by showing anti-carcinogenic properties, their interactions with EDCs remain underexplored in epidemiological studies and human clinical trials. On the other hand, some clinical investigations have evaluated the effects of polyphenols on endocrine-related endpoints, showing promising antioxidant and anti-inflammatory benefits for these compounds also in dysmetabolic/disendocrine conditions [187,188,189,190,191,192].

A recent technical review examined the relationship between soy and/or isoflavone intake and endocrine-related endpoints. In this review, which consisted of a comprehensive evaluation of 417 clinical studies, observational studies, systematic reviews, and meta-analyses (SRMA), it was concluded that the currently available evidence does not support classifying isoflavones as EDCs. The paper points out that, while isoflavones are endocrine-active, they do not meet the threshold to be classified as EDCs, even when taken at high concentrations (>100 mg/day), an important clarification that alleviates the public concern about soy intake [193].

Given the complexity of polyphenol-EDC interactions and the potential for polyphenols to exert both protective and harmful effects depending on the context and dosage, it is crucial to plan more targeted clinical trials. Future research should focus on evaluating the long-term consequences of polyphenol consumption on human health, as well as the synergistic effects of combining various polyphenols with dietary sources of EDCs. Of note, the effects of polyphenols rely on their bioavailability at the tissue level rather than on their intake. Gut flora, endogenous factors, and dietary variables can all impact bioavailability. Analyzing the synergistic effects of different polyphenols and their bioavailability is thus essential to optimize their potential therapeutic benefits while minimizing health risks. At the same time, a better understanding of the interplay between EDCs and polyphenols may allow for the rational design of improved approaches to protect human health from the risks associated with exposure to EDCs.

## Figures and Tables

**Table 1:** Protective effects of polyphenols against EDCs toxic effects.

Polyphenol	In Vivo Model	In Vitro Model	Effects	Reference
Resveratrol		Endometrial organoids from porcine uteri (10 µM)	Mitigation of BPA toxic effects via the modulation of Wnt/β-catenin	[95]
	Female adult albino Wistar rats (20 mg/kg BW/day, oral gavage)		Protection against BPA-induced uterine damage: restoration of normal gonadal hormone synthesis, reduction of oxidative stress, apoptosis and fibrosis	[96]
		Ovarian adenocarcinoma cell line (BG-1) (50–100 μM)	Reduction of cell growth induced by BPA, OP, MXC via upregulation of p21 expression and downregulation of CDK2 expression. Suppression of BPA-induced cell growth via disruption of ERα and IGF-1R pathways interaction	[104,105]
	Male albino Wistar rats (25, 50 mg/kg/day, i.p.)		Protection against BPA-induced metabolic abnormalities: improvement of insulin signaling and antioxidant capabilities	[137]
	Male albino Wistar rats (100 mg/kg, oral gavage)		Protection against BPA-induced damage	[138]
	Male Sprague-Dawley rats (100 mg/kg/day, oral gavage)		Reduction of the effects of BPA on testicular structure and sperm quality leading to higher levels of gonadotropin hormone and testosterone	[108]
	Male and female ApoE^−/−^ mice (20 mg/kg/day, oral)		Reduction of BPA-induced atherosclerotic alterations	[148]
	Virgin Sprague-Dawley (SD) rats (50 mg/L in drinking water throughout pregnancy and lactation)		Prevention of HF + BPA-induced hypertension: Restoration of NO availability, decrease of oxidative stress and AHR signaling	[144]
		Human CRC cell lines (HCT116, SW480) (100, 200 μM)	Prevention of Cd-induced migration and invasion through the control of the m6A alteration of ZEB1 and of EMT-related markers expression	[127]
		Cumulus oocytes complexes (COCs) from juvenile Sarda ewes (1 μM)	Mitigation of Cd-induced alterations, reduction of ROS accumulation, maintenance of the correct meiotic spindle and cortical F-actin assembly	[97]
	Male Hy-Line Variety White chickens (400 mg/kg, via diet)		Inhibition of Cd-induced kidney structural destruction and nephrotoxicity. Increased activity of antioxidant enzymes and reduction of Cd-induced oxidative stress. Reversal of Cd-induced mitochondrial ultrastructural alterations	[129]
		Osteoblastic Subclone14 cell line (MC3T3-E1) (5, 10 μM)	Improvement of osteoblast viability and early differentiation; osteoblasts protection from Cd damage	[143]
	Swiss Albino mice (20 mg/kg, oral)		Suppression of metal-induced perturbation of spermatogenesis, testicular morphology and the up-regulation of AKT cascade proteins and GCNIS markers	[107]
	Pregnant CD-1 mice (20 μM, via diet)		Decrease of Cd-induced placental toxicity by regulating DNMT3 expression and PI3K/Akt pathway activation	[98]
Resveratrol and Apigenin	Male Albino Wistar rats (60 days old, weight 200–350 g) (RES 100 mg/kg; apigenin 100 mg/kg, oral gavage)		Decrease of tissue oxidative stress and increase of tissue antioxidant levels. Protection against BPA-induced cytopathological alterations and apoptosis in salivary gland cells	[126]
Resveratrol, Curcumin, Bergamot juice (BJe)	Male C57 BL/6J mice (CUR 50,100 mg/kg; RES 20 mg/kg; BJe 40 mg/kg, oral)		Decrease of Cd-induced testicular damage via anti-inflammatory and anti-apoptotic mechanisms	[111]
Resveratrol Butyrate Ester (RBE)	15 weeks old pregnant female Sprague-Dawley rats (30 mg/kg/day, oral)		Decrease of BPA-induced oxidative damage in the liver; decrease of ALT and AST activities; increase of antioxidant enzymes expression and activity	[119]
	15 weeks old pregnant female Sprague-Dawley rats (30 mg/kg/day, gavage)		Decrease of BPA-induced weight gain and body fat accumulation. Increased blood concentration of lipid-related markers.	[139]
Curcumin		Transformed C3H Mouse Kidney-1 cells (TCMK-1) (10 μM)	Decrease of ATR-induced cell pyroptosis and cell cycle arrest via the reduction of oxidative stress	[132]
		Hepatic cell line (HepG2) (1, 2.5, 5 μM)	Decrease of BPA-induced insulin resistance	[140]
		Human normal cells (LO2) (10 μM)	Decrease of BPA-induced insulin resistance through the reduction of inflammation and block of JNK pathway	[141]
	Adult male albino rats (100 mg/kg/day, gavage)		Decrease of BPA-induced cardiac damage	[145]
	Adult female Wistar rats (20 mg/kg/day, i.p.)		Increase of adult hippocampal neurogenesis and correction of learning and memory deficits induced by BPA in AD models via the activation of the Wnt/B-catenin pathway	[134]
	Adult male Wistar rats (20 mg/kg, oral gavage)		Protection against BPA-induced neurotoxicity	[135]
	Adult male gerbils (*Meriones unguiculatus*) (100 mg/kg/day, oral)		Decrease of BPA harmful effects in the prostatic lobes	[115]
	Male C57BL/6 mice (200 mg/kg, oral gavage)		Decrease of ATR-induced endoplasmic reticulum stress and cardiac apoptosis	[54]
	Male Wistar rats (100 mg/kg/day, oral gavage)		Decrease of BPA-induced lipid peroxidation, inflammatory cells’ infiltration and necrosis in liver tissue	[121]
	Male CD-1 mice (0.5 mg/kg BPA and 0.1% *w*/*w* curcumin, via diet)		Prevention of BPA-induced hepatic steatosis through the limitation of intestinal absorption and hepatic cholesterol synthesis, reduction of liver cholesterol accumulation and improvement of liver lipid biosynthesis and fat accumulation	[123]
	Female Albino rats (200 mg/kg)		Reduction of the toxic effects of BPA on the glandular portions of the stomach, via anti-fibrotic and anti-apoptotic mechanisms	[128]
Curcumin and Quercetin	Adult Wistar rats (50 mg/kg curcumin, 50 mg/kg quercetin, oral gavage)		Curcumin enhances quercetin’s protective effects against ATR-induced testicular injury, increasing reproductive hormone levels, restoring testicular biochemical parameters	[110]
Quercetin	Male Sprague Dawley rats (50 mg/kg, oral gavage)		Restoration of spermatogenesis, reversal of histological damage induced by BPA, increase in plasma testosterone, estrogen decrease	[109]
	Adult male albino rats of Wistar strain (50 mg/kg, oral gavage)		Attenuation of Cd-induced biochemical alterations in serum, urine and renal tissue	[130]
EGCG	Male Swiss Webster mice (40 mg/kg, i.p.)		Suppression of DEHP-induced liver injury and AST activity; attenuation of DEHP-induced testis lesions, sperm deformity and spermatogenic cell apoptosis; reduction in CYP3A4 expression	[149]
EGCG and Green tea extract	Male albino Wistar rats (50 mg/kg green tea extract; 20 mg/kg EGCG)	HUVEC cell line (1, 2, 5, 10, 25, 50 μM)	Reduction of BPA-induced vascular dysfunction and atherosclerosis progression	[147]
*Eruca Sativa*		Human sperm cells (15.6 μg/mL)	Reversal of membrane potential and sperm motility changes induced BPA	[117]
Naringenin		Human breast cancer cell lines (MCF-7, T47D, MDA-MB-231) (1 nM–0.1 mM)	Inhibition of BPA-induced cell proliferation via the reduction of ERα(+) cells number and the prevention of BPA-induced AKT activation	[106]
Silymarin	Male CD-1 mice (200 mg/kg)		Protective effect against structural and ultrastructural injuries induced by BPA; reduction of pro-inflammatory cytokines levels	[120]
Lycopene	Male albino Wistar rats (10 mg/kg/day, oral gavage)		Improvement of BPA-induced alveolar collapse, lymphocytic infiltration, RBCs extravasation and fibrosis	[142]
	Female Wistar rats (10 mg/kg, oral gavage)		Reduction of BPA cytotoxic effects on hepatic tissues; improvement of liver function biomarkers and oxidant-antioxidant state, and reduction of DNA damage	[122]
Luteolin	Adult Wistar male rats (100–200 mg/kg, oral gavage)		Antioxidant effect, protection of the kidney from BPA-induced oxidative injury	[131]
Ginseng Extract	Adult female Albino rats (200 mg/kg, oral)		Decrease of phthalates and BPA reprotoxicity via the restoration of normal steroid hormone levels and the modulation of steroidogenic enzymes mRNAs	[103]
Isoflavones		Human neuroblastoma cell line (SH-SY5Y) (5 μM)	Increase of BEX2 expression, activation of BEX2-dependent autophagy and prevention of ATR-induced neuronal cell death	[136]

Abbreviations: AD, Alzheimer Disease; AHR, Aryl Hydrocarbon Receptor; ALT, Alanine Transaminase; ApoE, Apolipoprotein E; AST, Aspartate Transaminase; ATR, Atrazine; BEX2, Brain Expressed X-Linked Protein 2; BJe, Bergamot juice; BW, Body Weight; BPA, Bisphenol A; Cd, Cadmium; CDK2, Cyclin-dependent kinase 2; COCs, Cumulus oocytes complexes; CUR, Curcumin; DNMT3, DNA Methyltransferase 3; EMT, Epithelial-Mesenchymal Transition; ERα, Estrogen receptor α; GCNIS, Germ cell Neoplasia In Situ; HF, High Fat; i.p., intraperitoneal; JNK, c-Jun N-terminal kinases; MXC, methoxychlor; NO, Nitric Oxide; OP, Octylphenol; PI3K, Phosphatidylinositol 3 Kinase; RBC, Red Blood Cell; RBE, Resveratrol Butyrate Ester; RES, Resveratrol; ROS, Reactive Oxygen Species; SD, Sprague Dawley; TCMK-1, Transformed C3H Mouse Kidney-1; ZEB1, Zinc finger E-box binding homeobox.

**Table 2:** How polyphenols may enhance the detrimental effects of EDCs.

Polyphenol	In Vivo Model	In Vitro Model	Effects	Reference
		Human anaplastic thyroid carcinoma cell lines (HTh7, 8505C) (25, 50 μM)	Increased expression of thyroid-specific genes (Sodium/Iodide symporter (NIS) gene)	[183]
		Fisher Rat Thyroid cells (FRTL-5) (40, 50, 100 μM)	Increase of iodide influx	[184]
		F1 subclone of FRTL-5 rat thyroid cells (5, 10 μM)	Decreased expression of the sodium/iodide symporter and reduction of cellular iodide uptake	[185]
Resveratrol	Sprague-Dawley rats (25 mg/kg, i.p.)	F1 subclone of FRTL-5 rat thyroid cells (10 μM)	In vitro: decreased levels of Thyroid-specific genes (Thyroglobulin, Thyroid peroxidase, TSH receptor, Nkx2-1, Fox1, Pax8). In vivo: decrease of Thyroid proliferative activity	[186]
	Immunocompromised SCID mice with low metastatic ERα(−), ERβ(+) MDA-MB-231 and highly metastatic ER(−) MDA-MB-435 mammary tumors (5 mg/kg, oral gavage)		Promotion of breast tumor growth and metastasis. Increase of tumoral Rac activity	[181]
	Sprague-Dawley rats (100 mg/kg, oral gavage)	Leydig cells from 35-day-old Sprague-Dawley rats (100 μM)	Inhibition of LH-stimulated androgen synthesis in Leydig cells. Decrease of 3β-HSD activity in a competitive way	[179]
		Breast cancer cell lines (MCF-7, MDA-MB-231, T47D) (10 μM)	Inhibition of the binding of labeled estradiol to the ER and activation of the transcription of estrogen-responsive genes	[180]
		Trophoblast-derived Choriocarcinoma cell line (BeWo); Human first trimester placenta (HTR-O/SVneo) (from 0.1 μM to 1 mM)	Decrease of cell viability, BhCG secretion	[177]
Genistein	Outbred female CD-1 mice (50 mg/kg/day)		Increased risk of developing uterine adenocarcinoma	[174]
		Human prostate cancer cells (PC-3) (10, 50 μM)	Increased proliferation (<10 μM); cytotoxic effect (>10 μM) resulting in lower cells’ viability and migration	[164]
		Human cervical cancer cell line (HeLa) (10 μM)	Decrease of ZEN metabolites and potentiation of ZEN-induced toxicity	[162]
	Sprague-Dawley rats (100 mg/kg/day, uterine perfusion)		Increased uterine fluid secretion and accumulation, hyperplasia	[173]
Genistein and Quercetin		Breast cancer cell lines (MCF-7, SKBR3) (1 μM)	Increased c-fos expression	[156]
Quercetin		Granulosa cells isolated from ovaries of non-cycling pubertal gilts 180 days old (10 μg/mL)	Influence on basic ovarian activity (proliferation, apoptosis, steroidogenesis, FSH responsiveness)	[176]
	Bilaterally ovariectomized Sprague-Dawley rats (10 and 100 mg/kg/day, s.c.)		Affects uterine morphology and predisposes the uterus to tumor development	[172]
Daidzein	Pregnant DA/Han rats (10 mg/kg, i.v.)		Rapid transplacental transfer from mother to fetus	[171]
Isoflavone rich soy protein	Men at high risk for developing advanced prostate cancer (40 g/day, via diet)		Increased risk of developing advanced prostate cancer via the reduction of AR levels	[163]
Equol		Breast cancer cell line (MCF-7) (1 μM)	Increased cell proliferation	[170]

Abbreviations: 3β-HSD, 3β-Hydroxysteroid dehydrogenase; AR, Androgen Receptor; ER, Estrogen receptor; FSH, Follicle-Stimulating Hormone; i.p., intraperitoneal; LH, Luteinizing hormone; NIS, Sodium/Iodide Symporter; s.c., subcutaneous; SCID, severe combined immunodeficiency disease; TSH, Thyroid-stimulating hormone; ZEN, Zearalenone.

## Data Availability

Not applicable.
